# Balloon-Occluded Retrograde Transvenous Obliteration for Recurrent Hepatic Encephalopathy by the Trans-Paraumbilical Venous Approach

**DOI:** 10.7759/cureus.83549

**Published:** 2025-05-06

**Authors:** Hiroaki Ryo, Shuichiro Nakaminato, Naohiro Yamaya, Changwon Jeong, Eiki Obi

**Affiliations:** 1 Department of Radiology, Kameda Medical Center, Kamogawa, JPN

**Keywords:** balloon-occluded retrograde transvenous obliteration (brto), he: hepatic encephalopathy, liver cirrhosis (lc), portal hypertension, portosystemic shunts, trans-paraumbilical venous approach

## Abstract

Hepatic encephalopathy is a serious complication of liver cirrhosis often associated with portosystemic shunts (PSSs), which may be refractory to medical management. We present the case of a 58-year-old man with liver cirrhosis who developed recurrent hepatic encephalopathy associated with mesorenal and paraumbilical PSSs. Despite medical therapy, his symptoms recurred, necessitating interventional treatment. The first session consisted of partial splenic embolization and balloon-occluded retrograde transvenous obliteration (BRTO) of the mesorenal shunt via the transjugular route, resulting in transient improvement. Due to recurrence of encephalopathy, a second BRTO was performed via percutaneous trans-paraumbilical venous access under ultrasound guidance, allowing for stable catheterization and successful embolization with detachable coils, and partial splenic embolization was also performed during the second session to further control portal pressure. Following the procedures, the patient achieved sustained clinical and biochemical improvement, with no recurrence of encephalopathy during seven months of follow-up. This case highlights the safety and utility of the trans-paraumbilical venous approach as an alternative access route for BRTO in patients with complex PSSs.

## Introduction

Hepatic encephalopathy is a reversible alteration in consciousness, behavior, and psychomotor functions related to an accumulation of toxins like ammonia [1]. Causes of hepatic encephalopathy include liver cirrhosis, portosystemic shunts (PSSs), and metabolic disorders of the urea cycle. PSSs are present in approximately 60-63.5% of patients with liver cirrhosis [2,3]. Standard medical treatment for hepatic encephalopathy involves agents such as lactulose, rifaximin, and branched-chain amino acids (BCAAs). Nevertheless, these therapies are not always effective, particularly in refractory cases. For patients with portal hypertension and symptomatic PSSs, endovascular embolization using sclerosing agents, coils, or vascular plugs represents a recognized therapeutic option [4].

We present a case of hepatic encephalopathy due to a mesorenal and paraumbilical venous shunt. Two sessions of balloon-occluded retrograde transjugular obliteration (BRTO) were performed by the transjugular and trans-paraumbilical venous approaches. A percutaneous approach to the portal venous system via the paraumbilical vein has been reported [5-9]. However, it is not widely performed.

## Case presentation

A 58-year-old man with liver cirrhosis was transferred to our hospital with asterixis and bradykinesia. Laboratory examination revealed elevated serum ammonia at 170 µg/dL (reference range: 12-66 µg/dL). The patient’s Child-Pugh score was 9 (class B).

**Figure 1:**
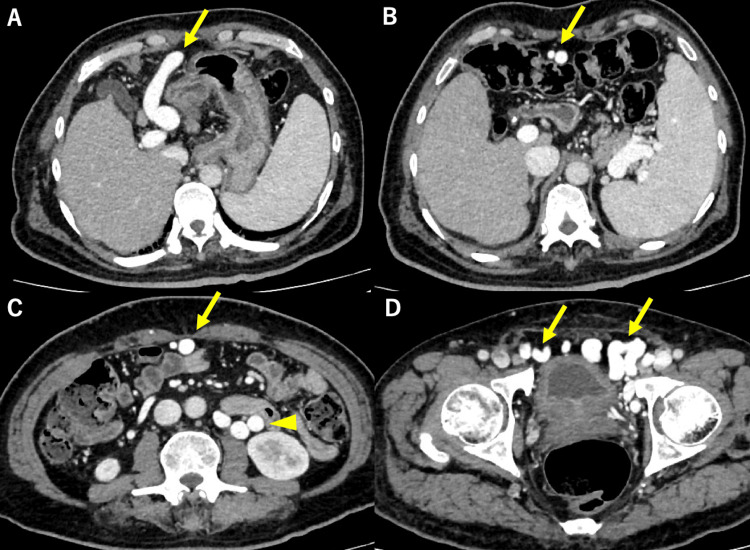
Contrast-enhanced CT of the portal phase. (A) Enlarged paraumbilical vein (yellow arrow) connecting the left branch of the portal vein. Splenomegaly is shown. (B) Paraumbilical vein running through the abdominal wall (yellow arrow). (C) In addition to the paraumbilical vein (yellow arrow), a mesorenal shunt is also seen (yellow arrowhead). (D) Paraumbilical vein connecting the bilateral common femoral vein (two yellow arrows).

Contrast-enhanced computed tomography (CECT) revealed cirrhosis, splenomegaly, and the two routes of the PSS: mesorenal shunt (MRS) and paraumbilical venous shunt connecting the left branch of the portal vein and the bilateral common femoral vein (Figure 1).

**Figure 2:**
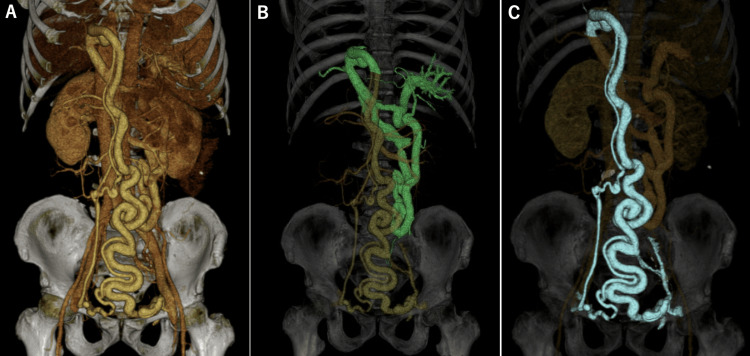
Three-dimensional volume-rendered CT images during the portal venous phase demonstrating two routes of the portosystemic shunt. (A) Overall view of both shunt pathways. (B) Mesorenal shunt (MRS), visualized in green. (C) Paraumbilical venous shunt extending from the left branch of the portal vein to the bilateral common femoral veins, visualized in blue.

A 3D-CT image of the portal venous phase demonstrated the two routes of the PSS mentioned above (Figure 2).

We diagnosed hepatic encephalopathy and initiated medical therapy, including rifaximin, BCAAs, and lactulose. Although his symptoms improved temporarily, they recurred two weeks later.

Therefore, we planned BRTO in two sessions. As the first session, to reduce portal hypertension, partial splenic embolization (PSE) with gelatin sponge particles was performed which was followed by BRTO.

**Figure 3:**
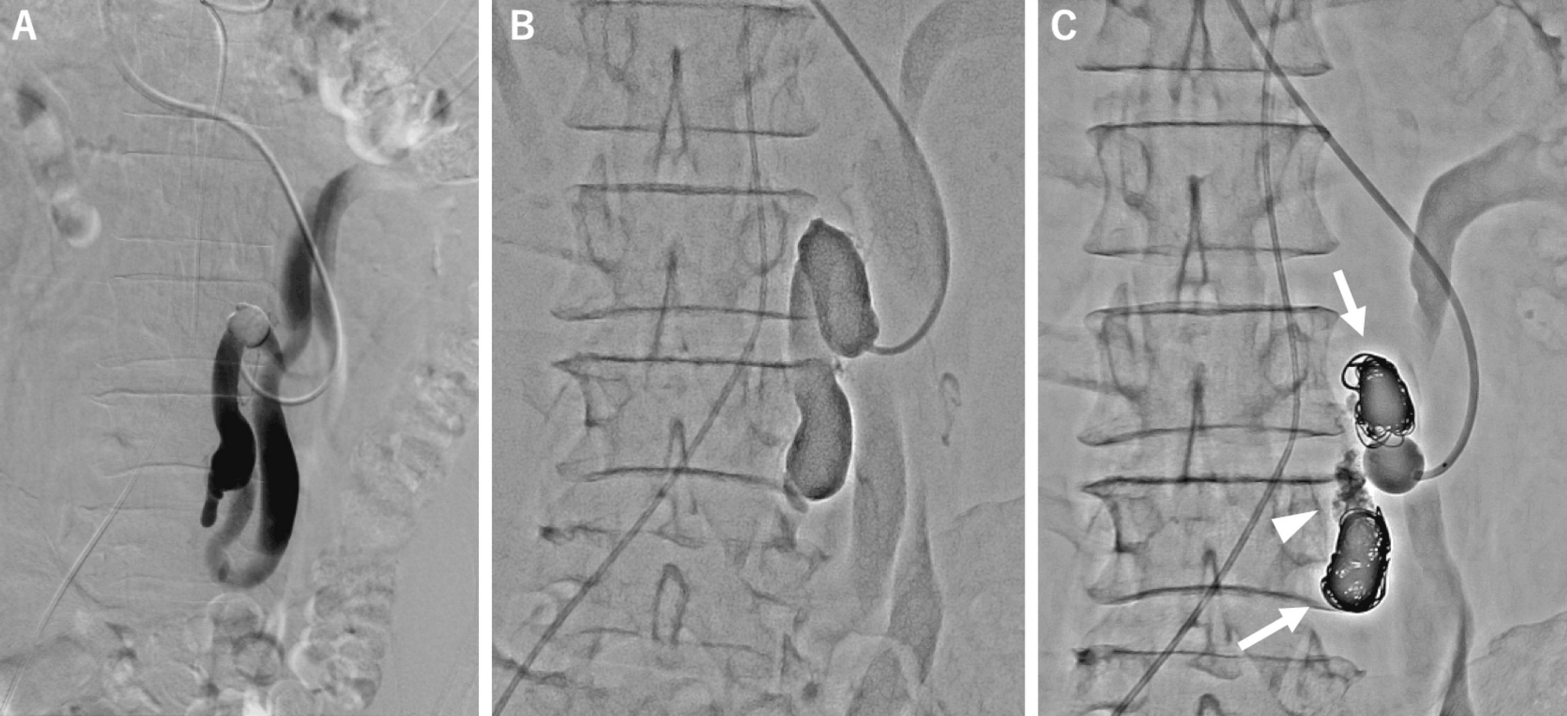
(A and B) An 8Fr sheath was inserted into the right jugular vein, and the tip of the sheath could be placed at the outlet of the mesorenal shunt (MRS) via the left renal vein. Balloon-occluded retrograde venography was performed with a 13mm balloon catheter which revealed overall anatomy of the MRS. (C) Embolization of the MRS. Arrows show the fibered coils. Arrowhead show the NBCA cast injected between coils. NBCA: N-butyl cyanoacrylate

An 8Fr sheath was inserted into the right internal jugular vein, and the tip of the sheath could be placed at the outlet of the MRS via the left renal vein. Balloon-occluded retrograde venography (BRTV) was performed with a 13mm balloon catheter (Selecon MP-Ⅱ, Terumo Clinical Supply, Gifu, Japan) which revealed overall anatomy of the MRS. MRS was embolized using detachable fibered coils (EMBOLD, Boston Scientific, USA). A 25% mixture of N-butyl cyanoacrylate (NBCA; Histoacryl, B. Braun, Melsungen, Germany) and Lipiodol (Guerbet, Paris, France) was injected between the distal and proximal coils (Figure 3).

After the procedure, CECT revealed thrombosis of the MRS. Twenty-two days after BRTO, his symptoms flared up with elevated serum ammonia (111 µg/dL). Therefore, additional embolization was planned via the paraumbilical vein.

**Figure 4:**
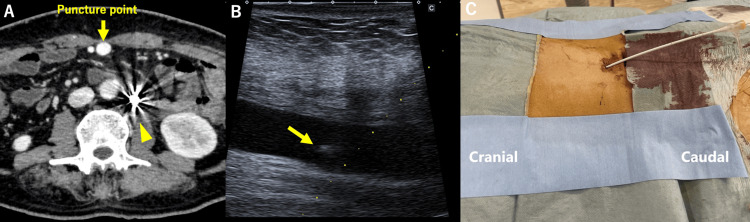
(A) CECT showed a dilated paraumbilical vein (yellow arrow), and MRS was well embolized using coils after the first BRTO (yellow arrowhead). (B) Paraumbilical vein was punctured with a 21G needle under ultrasound guidance. The yellow arrow shows the tip of the needle (yellow arrow). (C) A 7Fr sheath with a tip marker was inserted into the paraumbilical vein in the cranial direction. CECT: Contrast-enhanced computed tomography; BRTO: balloon-occluded retrograde transvenous obliteration; MRS: mesorenal shunt

As the second session, under local anesthesia, the paraumbilical vein was punctured percutaneously under ultrasound (US) guidance using a 21-gauge coaxial needle (Figure 4).

The 0.018-inch wire and 4Fr dilator in a percutaneous introducer system (AccustickⅡ, Boston Scientific, MA, USA) were used to introduce 4.5Fr sheath into the paraumbilical vein in the cranial direction (Figure 4). The 4.5Fr sheath was changed to a 7Fr sheath with a tip marker (Super sheath, Medikit, Tokyo, Japan).

**Figure 5:**
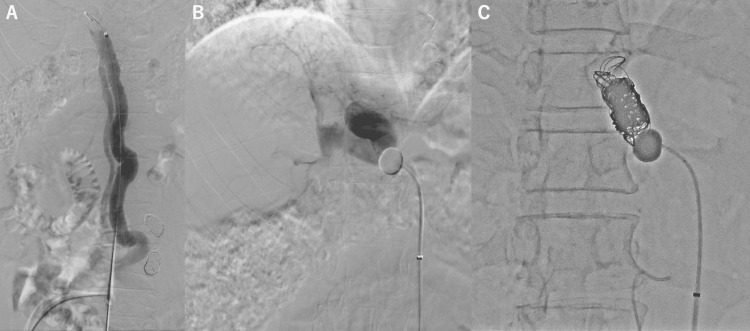
(A) Venography from upstream of paraumbilical vein. (B) BRTV revealed most of the intrahepatic portal vein. (C) Embolization of the upstream of the paraumbilical vein using coils. BRTV: Balloon-occluded retrograde venography

A 20mm balloon catheter (Selecon MP-Ⅱ, Terumo Clinical Supply, Gifu, Japan) was inserted between the intrahepatic portal vein and paraumbilical vein. BRTV revealed most of the intrahepatic portal vein. Portal venous pressure was 19 mmHg, and it was 31 mmHg with balloon occlusion. Paraumbilical vein was embolized using detachable fibered coils (Interlock and EMBOLD, Boston Scientific, MA, USA) (Figure 5).

**Figure 6:**
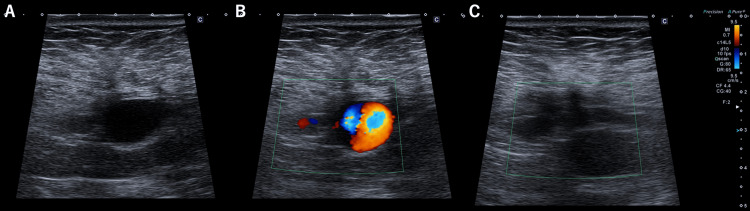
(A and B) US before BRTO showed dilated paraumbilical vein and flow signal was also clear. (C) After the second BRTO, the paraumbilical vein decreased in size and the blood flow signal disappeared. BRTO: Balloon-occluded retrograde transvenous obliteration

After the embolization, the paraumbilical vein decreased in size and the blood flow signal disappeared (Figure 6).

In addition, we performed PSE to reduce portal hypertension. Finally, the sheath inserted in the paraumbilical vein was removed and manual compression was applied for ten minutes.

**Figure 7:**
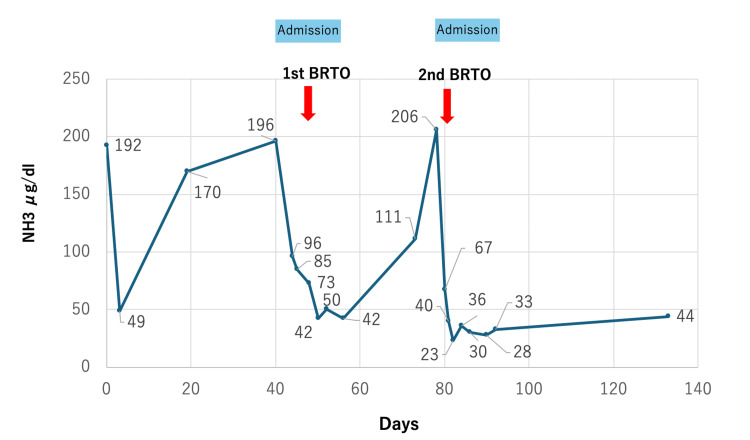
Clinical course of the present case. The serum ammonia ­(NH3) levels decreased after the second BRTO. BRTO: Balloon-occluded retrograde transvenous obliteration

Following the procedure, the patient showed symptomatic improvement, with a decrease in serum ammonia to 40 µg/dL (Figure 7).

**Figure 8:**
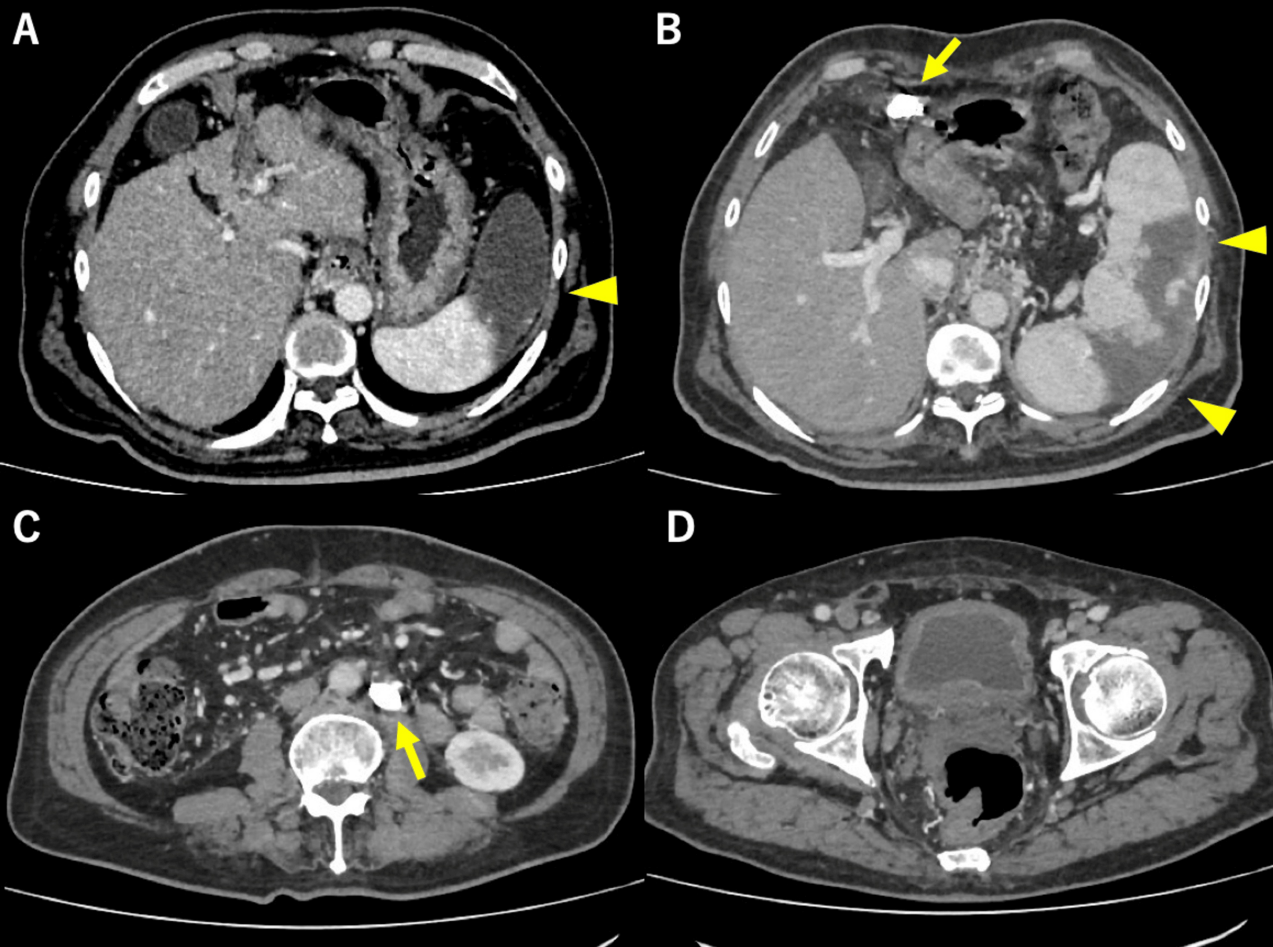
CECT after the second BRTO. Portosystemic shunts were embolized with coils (yellow arrows), and partial splenic embolization was performed (yellow arrowheads). (A) Partial splenic embolization is seen (arrowhead). (B) Coil in the paraumbilical vein (arrow) and splenic embolization (arrowheads). (C) Coil in the mesorenal shunt (arrow). (D) No residual shunts in the lower abdomen. CECT: Contrast-enhanced computed tomography; BRTO: balloon-occluded retrograde transvenous obliteration

Follow-up CECT revealed that each PSS was well embolized and approximately 60% of the spleen was embolized; however, thrombus in the left branch of the portal vein developed and ascites increased. Following the administration of the oral anticoagulants and diuretics, the thrombus decreased, and the ascites disappeared (Figure 8).

The patient’s Child-Pugh score was improved from 9 to 6 (class A). The patient was discharged home 15 days after the second procedure. No recurrence of hepatic encephalopathy was noted during a seven-month follow-up.

## Discussion

We report the case of recurrent hepatic encephalopathy treated by BRTO using a transjugular and trans-paraumbilical venous approach. Spontaneous recanalization of blood ﬂow in the umbilical vein has been reported to occur in 26% of patients with cirrhosis of the liver and portal hypertension [5]. The use of the percutaneous trans-paraumbilical venous approach was reported for esophageal varix embolization more than four decades ago [6]. Although there have been some reports of ectopic varix embolization with this approach, it is not widely used [7-10].

Kamada et al. reported cases of embolization using the trans-paraumbilical venous approach for the treatment of hepatic encephalopathy [11,12]. They also described that the advantages of the trans-paraumbilical venous approach are its accessibility to the target vessel and stability of the catheter system. Furthermore, the paraumbilical vein can be safely punctured under US guidance [9]. Published reports have indicated that puncturing the anterior wall of the umbilical vein may help prevent intra-abdominal hemorrhage [8,11].

In fact, in our case, we successfully punctured the paraumbilical vein on the first try. Moreover, we achieved adequate coil embolization due to the stable backup force. If the paraumbilical venous shunt were approached from the femoral vein, the catheter could fail to reach the shunt or result in incomplete embolization.

In the case of paraumbilical venous approach, the risk of bleeding from the puncture site is a concern, even in patients with coagulopathy and decreased liver function (Child-Pugh class C); manual compression of the puncture site for ﬁve minutes can lead to hemostasis [9]. Although hemostasis of the puncture site was obtained in our case, it is ideal to assess the flow signal by US before the system is removed.

After BRTO, we need to consider the following points. Refractory ascites, esophageal variceal rupture, and hepatic failure have been reported as complications related to increased portal pressure following shunt embolization [13,14]. The occurrence of esophageal varices is more frequent in groups with portal pressure measurements higher than 30 mmHg before BRTO [15]. In our case, the portal pressure before BRTO was 19 mmHg, which was not so high, but after BRTO, the patient had increased ascites and required diuretics. Although embolization of multiple shunts in a single session has been reported, it is often prolonged and technically complex and may lead to rapid portal hypertension, increasing the risk of complications such as refractory ascites and ectopic varices. It is also technically difficult to treat multi-PSSs in one session because of the time required. Therefore, we conducted BRTO in two sessions. Waguri et al. indicate that concomitant PSE may help diminish the increase in portal venous pressure after B-RTO for portosystemic shunts [16]. Therefore, we added PSE with BRTO, but the prolonged procedure time, increased amount of contrast media, and pain control after PSE are often problems.

## Conclusions

In this case, BRTO was performed in two separate sessions to treat mesorenal and paraumbilical PSSs. The trans-paraumbilical venous approach provided direct and stable access to the target vessel, enabling effective embolization. This technique proved to be both feasible and safe and may serve as a valuable alternative when conventional routes are not suitable.
